# Results of a Web-Based Survey on 2105 Greek Migraine Patients—Second Part: Efficacy of Acute and Prophylactic Migraine Treatments and Corresponding Patients’ Reported Satisfaction

**DOI:** 10.3390/medicina59010031

**Published:** 2022-12-23

**Authors:** Emmanouil V. Dermitzakis, Aikaterini Kouroudi, Andreas A. Argyriou, Konstantinos C. Spingos, Konstantinos Bilias, Michail Vikelis

**Affiliations:** 1 Euromedica General Clinic, 54645 Thessaloniki, Greece; 2 Greek Society of Migraine and Headache Patients, 11743 Athens, Greece; 3 Neurology Department, Agios Andreas State General Hospital of Patras, 26335 Patras, Greece; 4 Corfu Headache Clinic, 49150 Corfu, Greece; 5 Headache Clinic, Mediterraneo Hospital, 16675 Glyfada, Greece

**Keywords:** migraine, chronic migraine, web-based survey, COVID-19 pandemic, preventive treatment, symptomatic treatment, efficacy, patients’ satisfaction

## Abstract

*Background and Objectives:* The Greek Society of Migraine and Headache Patients conducted, in 2020, its second online survey, titled “Migraine in Greece—2020”, after publication of the first similar online survey conducted in 2018. To compare the current findings with the corresponding data obtained in 2018, we herein release the second part of results obtained from the 2020 survey on the efficacy of preventive and symptomatic anti-migraine medications and the patients’ reported satisfaction with these treatments. *Materials and Methods:* We surveyed 2105 migraine patients from all over Greece with the use of a 151-questions specific migraine-focused questionnaire in Greek language, which was distributed through the online research software “SurveyMonkey”. *Results:* Triptans were mostly used with efficacy for the symptomatic relief of migraine attacks. About 2 of 3 surveyed patients had received various prophylactic oral medications and the majority of them discontinued these prophylactic medications as a result of inefficacy/safety issues. BoNTA was reported to be effective only when administration was commenced by a trained neurologist/headache specialist, while our current findings are generally comparable to those obtained in our 2018 pre-COVID-19 survey and the pandemic has not imposed any significant attitudes on migraine therapies and corresponding patients’ satisfaction. *Conclusion:* Although a market change is anticipated with the evolving widespread use of anti-CGRPs monoclonal antibodies or gepants in the symptomatic and prophylactic treatment of migraine, it is of great interest to review published results of larger longitudinal population-based studies to further ascertain the satisfaction of patients to migraine therapies.

## 1. Introduction

Migraine, clinically classified as either episodic (low frequency episodic: 4–7 days monthly; high frequency episodic: 8–14 days monthly) or chronic migraine (more than 15 headache days monthly, of which at least 8 are of migrainous type for more than 3 months), is a common primary headache disorder imposing a significant burden on patients [1]. Despite chronic migraine (CM) being considered to be much more disabling compared to episodic migraine (EM) [2], there is evidence from a study collecting data from American Migraine Foundation’s (AMF) American Registry for Migraine Research (ARMR) that the current definition of migraine, based on the number of monthly headache days, may not reflect the exact differences in burden and disability among EM and CM patients, as high frequency EM patients experience a comparable degree of disability with CM patients having 15–23 monthly headache [3].

There are significant unmet clinical needs in relation to migraine acute treatment, but mostly with its prophylaxis. Several oral non-specific preventative medications are currently used as first line treatments in both EM and CM, including antihypertensives, antiepileptic drugs, beta and calcium channel blockers, and various antidepressants [4]. However, there is robust evidence to support that the use of these pharmacological preventive approaches is modestly effective, coupled with poor long-term adherence and a significant percentage of treatment discontinuations after 12 months of treatment, due to safety/tolerability issues [5]. The release of injectable preventatives with the use of anti-calcitonin gene-related peptide (CGRP) monoclonal antibodies or onabotulinumtoxin A elevated the expectations for a much-improved efficacy/tolerability ratio in migraine prophylaxis [6,7,8]. Concerning the symptomatic treatment of acute attacks with triptans, the same drawbacks occur, as high rates of medication overuse headache occur with triptan overuse [9,10].

Although previous population-based observational studies conducted with the use of Internet- or telephone-led surveys from USA or Europe [11,12,13,14] have shed light on the demographic and epidemiological data, severity, and impact of the disease in migraine sufferers, there are still issues to be further explored mainly in relation to the access to treatment and satisfaction to available medications based on patients’ level of disability, particularly during the outbreak of the COVID-19 pandemic. On a national Greek level, it was evident from the results of an observational descriptive survey, using computer-assisted telephone interviews, that although migraine is the most common primary disabling headache disorder in Greece, the majority of migraine participants were not under any preventative treatment [15].

Globally, there are associations of patients with migraine that aim to raise public and state awareness on headaches and treatment options through publication of results from population web-based surveys. Towards the latter view, the non-profit Greek Society of Migraine and Headache Patients (GSMHP), which was established in 2017, conducted in 2020 (year of COVID-19 pandemic) its second online survey, titled “Migraine in Greece—2020”, after publication of the first similar online survey in 2018. The 2020 web-based survey included 2105 migraine patients, who were called to answer 151 questions, covering the whole spectrum of demographics, clinical phenotype, and treatment of patients with migraine.

We recently published the first part of results concerning the demographic and clinical characteristics of participants, the severity and effects of migraine on their quality of life, and the effects of the coronavirus pandemic on the course of migraine [16]. In an attempt to compare the current findings with the corresponding data obtained from a similar survey conducted by GSMHP in a sample of 1091 migraine patients in 2018 [17] and the results by Constantinidis et al. [15], which both concluded that migraine remains both underdiagnosed and undertreated in Greece, we herein release the second part of results obtained from the 2020 population-based survey on the efficacy of preventive and symptomatic anti-migraine medications as well as the patients’ reported satisfaction with these treatments.

## 2. Materials and Methods

The data collection occurred over a period of two months from June 1 to July 31 2020. The methods for conducting this survey are described in detail in our recent publication containing the first part of its results concerning the demographic and clinical characteristics of participants, the severity and effects of migraine on their quality of life, and the effects of the coronavirus pandemic on the course of migraine [16]. The same methods were used in the current second part of results on the efficacy of preventive and symptomatic anti-migraine medications and the patients’ reported satisfaction with these treatments.

Briefly, candidate participants from all over Greece (all 13 regions) were randomly called to reply to a 151-questions specific migraine-focused questionnaire in Greek language, which was distributed through the online research software “SurveyMonkey”. The target group included GSMHP members (personal email invitation), but not exclusively, as the survey was promoted through GSMHP’s social media accounts (Facebook, Instagram, and Twitter). In 2020, GSMHP formally had 1812 members and 10,000 followers in its social media accounts. Eventually, this survey included adult patients with a definite diagnosis of migraine in a two-stage procedure, i.e., (i) the screening interview (first stage), wherein demographics and residency were first collected and then participants had to reply to a screening question expressed as “do you have a definite diagnosis of migraine established by a physician or do you have clinical symptoms resembling migraine but not formally diagnosed by a physician?”, whereas afterwards, they completed key clinical questions to ascertain whether the diagnostic criteria for a definite migraine diagnosis were fulfilled [18]; (ii) participants failing to fulfil the migraine diagnostic criteria were not allowed to proceed further in the online survey (second stage), in which only definite migraineurs were able to continue and complete all primary questions in relation to the efficacy of preventive and symptomatic anti-migraine medications as well as the patients’ reported satisfaction to these treatments. At the end of the procedure, patients were allocated in two groups: those with a diagnosis of migraine established by a physician (Group A), and cases with clinical migraine symptoms who had not been diagnosed by a medical professional (Group B). The flow diagram of participants remained the same as previously presented [16].

The study was performed in accordance with the principles of the Declaration of Helsinki, while its protocol was approved from the ethics committee of Euromedica General Clinic, Thessaloniki, Greece. At the beginning of the study procedure, each patient had to read a mandatory consent form so as to be informed about the objectives, which was in agreement with regulations of data protection, and clearly note her/his agreement in order to be further allowed to anonymously complete the web-based survey.

### Statistical Analysis

Descriptive statistics, performed with the SPSS for Windows (release 27.0; SPSS Inc., Chicago, IL), generated categorical variables (observed counts and weighted percentages) and continuous variables (mean or median with the corresponding standard error or range), depending on their nature. The Mann–Whitney U test for two samples was used for non-parametric comparisons, whereas the Chi-square one-sample test and Chi-square with Yates corrected *p*-value were used to compare proportions. Significance was set at the *p* < 0.05 level.

## 3. Results

As previously mentioned, the study enrolled 2105 patients who after completion of the first diagnostic part of the questionnaire fulfilled the diagnostic criteria for migraine, according to both the ICHD-3 symptom criteria [18] and physician diagnostic criteria. The baseline epidemiological and clinical characteristics of participants were previously described in detail [16]. Briefly, we surveyed 159 males (7.6%) and 1946 females (92.4%) with a mean age of 32.5 ± 14.3 (range: 18–60) years, who failed to a median number of 3 (range: 0–7) previous prophylactic medications. Most common comorbidities included various chronic pain syndromes (*N* = 286; 58.8%), followed by hypo/hyperthyroidism (*N* = 211; 43.4%). A total of 1248 (83.5%) participants had EM, either very low, low or high frequency, while 247 (16.5%) were with CM. Among the total of 2105 participants, 1550 of them had been diagnosed by a physician (group A), and 555 cases were allocated to group B as they were at baseline with clinical migraine symptoms but lacked a formal migraine diagnosis established by a medical professional. Group B patients were without any other evidence of suffering from another type of headache disorder.

We obtained the following data regarding the efficacy of preventive and symptomatic anti-migraine medications and the patients’ reported satisfaction to these treatments.

### 3.1. Prophylactic Anti-Migraine Treatments (Group A Only)

**Figure 1:**
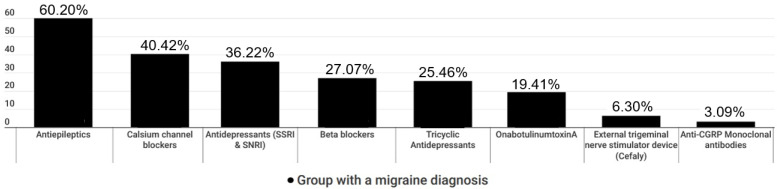
Prophylactic migraine treatments only in patients with a migraine diagnosis by a physician (group A).

Among patients with established migraine diagnosis by a physician, 815 patients (57.23%) had received various prophylactic treatments. The answers to the multiple-choice question “Which prophylactic treatment(s) have you received at least once are shown (drug class)?” is described in Figure 1.

Nonetheless, the majority of participants (64.2%) prioritized safety over effectiveness in medications prescribed for their migraine prophylaxis.

#### 3.1.1. Antiepileptics

A total of 490 (60.20%) of the responders had received antiepileptics for their migraine prophylaxis, either topiramate and/or valproic acid. Of those, 139 (28.95%) were not happy at all with this particular prophylaxis, 151 (30.81%) were modestly happy, 113 (23.06%) were fairly happy, 43 (8.77%) were very happy, and 28 (5.71%) were extremely happy. When asked “Did you stop taking antiepileptics for some reason?”, 345 (70.4%) answered positively. Of those, 227 (65.8%) stopped due to ineffectiveness and 190 (55.07%) because of side effects such as weight loss or gain, agitation, dizziness, somnolence, numbness, and clumsiness or unsteadiness.

#### 3.1.2. Antidepressants

In total, 503 (61.8%) participants had been treated with antidepressants as a preventive treatment, 182 (36.3%) with SSRIs or SNRIs (duloxetine, venlafaxine) and 128 (25.5%) with tricyclics (amitriptyline). Of those, 113 (22.46%) were not happy at all with the corresponding effects, 151 (30.02%) were modestly happy, 104 (20.67%) were fairly happy, 45 (8.94%) were very happy, and 19 (3.77%) were extremely happy. To the question “have you stop antidepressants for some reason?”, 273 (54.27%) answered positively. Among them, 190 (63.59%) stopped the antidepressants due to ineffectiveness and 146 (53.47%) due to side effects such as weight gain, agitation, dizziness, somnolence, and indigestion/stomach aches.

#### 3.1.3. Beta Blockers or Calcium Channel Blockers

Prophylactic treatments with either beta blockers (propranolol) or calcium channel blockers (flunarizine) were commenced in 221 (27.07%) and 329 (40.42%) patients, respectively. The vast majority (90%) of propranolol-treated patients discontinued treatment due to side effects, such as bradycardia, irritability, and mood disorders, while ineffectiveness was the main cause of flunarizine intake, coupled with weight gain as a side effect.

#### 3.1.4. OnabotulinumtoxinA (BoNTA)

Among the 158 (19.41%) responders with CM who had been treated with BoNTA, only 99 (62.65%) answered that their treatment was performed with the approved fixed site-fixed dose PREEMPT protocol by a trained neurologist. The remaining 59 patients received BoNTA by dermatologists or ENT physicians at arbitrary and unapproved injection sites, i.e., just in forehead, and dosage. To the question “How happy are you with the effect of BoNTA treatment in migraine prophylaxis”, 42 (26.58%) were not happy at all (37 of them were treated by a non-neurologist physician), 57 (36.07%) were modestly happy, 30 (18.98%) were fairly happy, 15 (9.49%) were very happy, and 12 (7.59%) were extremely happy. The question “Have you stopped your treatment with BoNTA for any reason?” was answered positively by 87 (55.06%) of the responders. When asked (multiple choice) “Why did you stop your treatment with BoNTA?”, 83 (95.4%) responded “due to ineffectiveness” and 4 (4.6%) responded “due to side effects”, including neck pain and eyelid ptosis.

#### 3.1.5. External Neurostimulation Devices (Cefaly^®^)

Among migraine responders that had been treated with the only readily available in Greece external neurostimulation device Cefaly^®^ (*N* = 51; 6.3%), the question “How happy are you with the way you are treating your migraine with Cefaly^®^” was answered as “not happy at all” by 23 (45.1%), “modestly happy” by 13 (25.49%), “fairly happy”, by 14 (27.45%), “very happy” by 0 (0%), and “extremely happy” by 1 (1.96%). When asked “Have you stopped treatment with Cefaly^®^ for any reason” 38 (74.5%) answered positively. A total of 35 (92.10%) of those discontinued “due to lack of effectiveness” and 3 (7.9%) “due to side effects” (both answers were allowed).

#### 3.1.6. Anti-CGRP Monoclonal Antibodies

A relative low number of patients (25, 3.09%) had received anti-calcitonin gene related peptide (CGRP) monoclonal antibodies (anti-CGRP MAbs), because in Greece, fremanezumab was approved for reimbursement by the Greek National Health System (NHS) and social services on July 2021 and erenumab followed on February 2022 (both approval dates occurred after finalization of this survey). Anti-CGRP MAbs-treated participants were questioned (multiple choice) “How did you learn about the new anti-CGRP MAbs treatment?”. The majority of them (17, 68.0%) responded “by my doctor”, 6 (24.0%) “by the GSMHP” and 2 (8.0%) “through the Internet”. When asked “How happy are you with the way you are treating your migraine with the anti-CGRP MAbs?” 2 (8%) answered “not happy at all”, 7 (28%) “modestly happy”, 4 (16%) “fairly happy”, 4 (16%) “very happy”, and 8 (32%) “extremely happy”. The question “Have you stopped treatment with monoclonal antibodies for any reason?” was answered negatively by 15 (60.0%), and positively by 10 (40.0%). When asked (multiple choice) “Why did you early stop taking anti-CGRP MAbs” 5 (50.00%) answered “due to high cost”, 4 (40.00%) “due to lack of effectiveness” and 1 (10.00%) “due to side effects”.

#### 3.1.7. Complementary and Alternative Medicine Treatments

When participants of both groups A and B were asked “Have you tried at least once some kind of prophylactic complementary and alternative medicine treatment (CAMT; homeopathy, acupuncture, supplements, nutrients, etc.)?”, 1032 (49.02%) of them answered negatively and 1073 (50.98%) answered positively. Of those 1073 participants, 404 (37.65%), 337 (31.4%), 254 (23.66%), and 78 (7.27%) had tried acupuncture, homeopathy, dietary supplements, and reflexology, respectively. (Question (multiple choice): “Which types of prophylactic CAMT treatment have you tried at least once?”).

A total of 165 (40.84%), 118 (29.20%), 80 (19.80%), 26 (6.43%), and 15 (3.71%) participants were “not happy at all”, “modestly happy”, “fairly happy”, “very happy” and “extremely happy”, respectively, with acupuncture. (Question “How happy are you with the way you are treating your migraine with acupuncture?”). A total of 335 (82.92%) participants stopped acupuncture due to ineffectiveness. (Question “Have you stopped your acupuncture treatment for any reason?”).

In all, 120 (35.60%), 124 (36.79%) 49 (14.54%), 27(8.01%), and 17 (5.04%) participants were “not happy at all”, “modestly happy”, “fairly happy”, “very happy”, and “extremely happy”, respectively, with homeopathy. Question: “How happy are you with the way you are treating your migraine with homeopathy?”. In total, 273 (81.0%) participants had stopped homeopathy due to ineffectiveness. Question: “Have you stopped your homeopathy treatment for any reason?”.

Among 254 patients using dietary supplements and nutrients, 180 (70.87%), 41 (16.10%), 29 (11.40%), and 4 (1.57%) used magnesium, vitamin complexes, cannabidiol products, or another supplement, respectively. (Question (multiple choice) “Which dietary supplements do you use?”). In the responses, 69 (27.16%), 110 (43.3%), 54 (21.25%), 19 (7.48%), and 2 (0.78%) participants were “not happy at all”, “modestly happy”, “fairly happy”, “very happy”, and “extremely happy” with a dietary supplement, respectively. (Question “How happy are you with the way you are treating your migraine with dietary supplements?”). A total of 130 (51.18%) participants had stopped dietary supplements due to ineffectiveness. (Question “Have you, for some reason, stopped using dietary supplements?”).

### 3.2. Acute Migraine Treatment (Group A and B)

**Figure 2:**
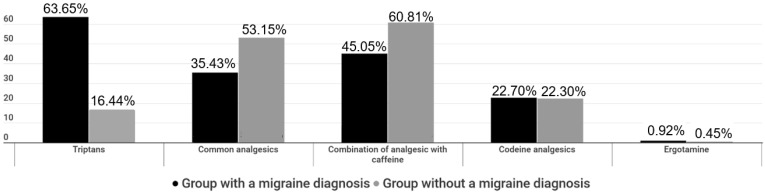
Acute symptomatic migraine treatments in both group A and B patients.

In the sample, 1428 (95.71%) participants from group A and 450 (90.36%) from group B systematically used symptomatic treatment. (Question “Do you receive symptomatic treatment (painkillers) during crisis?”). The answers to the multiple-choice question “During migraine attacks, what symptomatic treatment do you receive?” are shown in Figure 2.

When asked “How long after the onset of migraine crisis do you receive your symptomatic treatment?”, 883 (62.45%), 373 (26.38%), and 158 (11.17%) group A patients as well as 216 (48.65%), 117 (26.35%), and 111 (25.00%) group B patients answered “20 min”, “1 h”, and “over 1 h”, respectively. To the question “How many days per week, on average, have you received any acute symptomatic treatment during the last trimester?”, 812 (57.43%), 487 (34.44%), and 115 (8.13%) group A patients and 339 (76.35%), 93 (20.95%), and 12 (2.70%) group B patients answered “6–7 times”, “0–2 times” and “3–5 times” per week, respectively. When asked “How many pills per week did you take, on average, to treat migraine during the last trimester?”, 517 (36.56%), 456 (32.25%), 288 (20.37%), and 153(10.82%) group A patients and 221 (49.77%), 135 (30.41%), 63 (14.19%), and 25 (5.63%) group B patients answered “0–2”, “3–4” “5–8” and “over 8” pills per week, respectively.

#### 3.2.1. Simple Analgesics and Non-Steroidal Anti-Inflammatory Drugs (NSAIDs)

In all, 618 (43.99%) patients of group A and 281 (63.43%) patients of group B reported systematic consumption of various simple analgesics and NSAIDs, such as paracetamol, ibuprofen, and naproxen. When asked “How happy are you with the way you are treating your migraine with simple analgesics and NSAIDs”, 87 (14.12%), 285 (46.27%), 204 (33.12%), 32 (5.19%), and 8 (1.30%) group A patients and 23 (8.16%), 114 (40.43%), 118 (41.84%) 20 (7.09%), and 7 (2.48%) group B patients were “not happy at all”, “modestly happy”, “fairly happy”, “very happy”, and “extremely happy”, respectively. Nonetheless, 365 (59.25%) group A patients and 199 (70.57%) group B patients continued taking simple analgesics and NSAIDs for symptomatic relief of acute migraine attacks. (Question “Have you stopped taking simple analgesics for some time for any reason?”). When asked (multiple choice) “Which was the reason(s) for discontinuing intake of simple analgesics and NSAIDs” 232 (92.06%) group A patients as well as 76 (93.83%) group B patients answered “due to ineffectiveness”.

#### 3.2.2. Combination of Paracetamol with Caffeine

A total of 660 (47.76%) patients from group A and 255 (59.03%) patients from group B reported using some combination of paracetamol and caffeine. When asked “How happy are you with the way you are treating your migraine with a paracetamol/caffeine combination?”, 28 (4.24%), 220 (33.28%) 322 (48.71%), 64 (9.68%), and 27 (4.08%) group A patients and 5 (1.98%), 64 (25.40%), 137 (54.37%), 34 (13.49%), and 12 (4.76%) group B patients answered “not happy at all”, “modestly happy”, “fairly happy”, “very happy”, and “extremely happy” with simple analgesics, respectively. When asked “Have you stopped taking an analgesic/caffeine combination for any reason?”, 510 (77.16%) group A patients and 202 (80.16%) group B patients answered negatively, respectively. When asked (multiple choice) “Why did you stop taking a paracetamol/caffeine combination?” 111 (73.51%) and 50 (33.11%) group A patients and 35 (70.0%) and 20 (40.0%) group B patients answered “due to ineffectiveness” and “due to side effects”, respectively.

#### 3.2.3. Triptans

Triptans use was reported by 850 patients from group A and 79 patients from group B (61.06% vs. 18.08%, respectively; *p* < 0.001). When asked “How happy are you with the way you are treating your acute migraine with triptans?”, 20 (2.35%), 96 (11.28%), 359 (42.19%), 247 (29.02%), and 129 (15.16%) group A patients and 1 (1.25%), 13 (16.25%), 38 (47.50%), 14 (17.50%), and 14 (17.50%) group B patients answered, “not happy at all”, “modestly happy”, “fairly happy”, “very happy”, and “extremely happy” with simple analgesics, respectively. When asked “Have you stopped taking triptans for any reason?” 658 (77.32%) group A and 74 (92.50%) group B patients answered negatively. To the multiple-choice question “Why did you stop taking your triptan?”, 118 (61.14%) and 97 (50.26%) group A patients and 4 (57.14%) and 3 (42.86%) group B patients answered “due to side effects” and “due to ineffectiveness”, respectively.

#### 3.2.4. Complementary and Alternative Medicine (CAM) as Acute Treatment of Migraine Attacks

**Figure 3:**
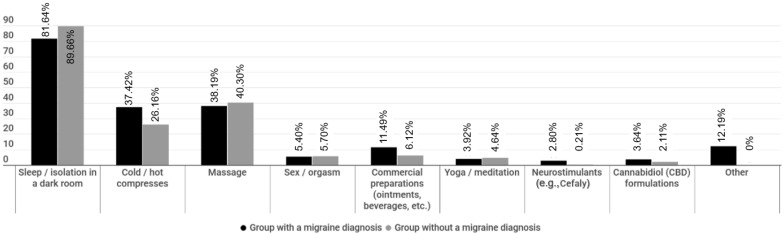
Alternative acute migraine treatments in both group A and B patients.

The use of CAM was reported by 1427 group A and 474 group B patients. The answers of the two groups to the multiple-choice question “What alternative symptomatic treatment do you use to relieve your migraine attack in case that you do not use conventional medical treatment?” are shown in Figure 3.

### 3.3. Associations

When we examined potential associations of demographic and baseline clinical characteristics with both acute and prophylactic treatment preferences, we found gender effects with males favoring CAM approaches three times more than females (odds ratio (OR) 2.9, *p* < 0.001), compared to pharmacological approaches. Age effects were also noted in relation to the acute and prophylactic pharmacological medication intake, which was much lower in the 18–35 age group, compared to older patients, especially to those in the 45–59 age group (OR 1.5, *p* = 0.01). We were not able to detect any other notable effect of demographic and baseline clinical characteristics on treatment preferences.

## 4. Discussion

With the release of the second part of results from the current population web-based survey conducted by GSMHP in 2020, we primarily aimed to trace potential treatment differences of patients with migraine in Greece, according to study groups (group A: migraine diagnosed by a physician; group B: with clinical migraine symptoms at baseline but lacked a formal migraine diagnosis established by a medical professional), focusing on the assessment of efficacy of preventive and symptomatic anti-migraine medications as also the satisfaction of patients to these treatments, in terms of safety/efficacy. In addition, we sought to compare the relevant results of the 2020 survey with those previously obtained in 2018 [17].

Our results, overall, point towards the view that patients whose migraine was diagnosed by a neurologist/headache expert (group A) had higher disability and were more difficult to treat than patients belonging to group B, a fact which most likely contributed to ask specialized consultation. Amongst formally diagnosed migraine patients (group A), 815 (57.23%) have received prophylactic anti-migraine treatment with orally and/or injectable-administered medications, a percentage that is comparable to the corresponding findings of the 2018 survey in which 58.2% of participants received various prophylactic treatments [17]. This consistency in percentages of patients under anti-migraine prophylaxis between timepoints (2018 vs. 2020) shows that about 40% of migraineurs remain untreated, despite the current raised awareness and improved migraine care; however, it should be mentioned that a small percentage of them was with very low frequency episodic migraine and no need for prophylaxis. Nonetheless, it is also evident that more than 50% of patients experience migraine intensification during the course of the disease, in terms of both frequency and intensity, which is significant enough to impose disability such that patients seek consultation by a neurologist/headache expert and are eventually treated with available prophylactic treatments.

Specifically for migraine prophylaxis with the use of antiepileptic or antidepressant medications, it was evident that about 60% of 815 participants from group A had received either topiramate/valproic acid or SSRIs/SNRIs/tricyclics, with modest efficacy/safety as the majority of them (61%) remained null to minimally satisfied from these medications due to inefficacy (about 70%) or evidence of side effects (about 55%). Very or quite satisfied from antiepileptics/antidepressants only represented 15% of our interviewed patients. About 65% (2 out of 3) of our participants discontinued these prophylactic medications as a result of inefficacy/safety issues, findings which are in close agreement with data demonstrating that up to 55% of migraine patients discontinue oral pharmacological preventive approaches after 12 months of treatment [19], because of modest response, poor adherence, and compliance to more than one line of such prophylactic therapies [20]. Interestingly, amitriptyline was associated with the higher rates of satisfaction due to efficacy among antidepressants, thoroughly demonstrating that it either has direct antimigraine prophylactic properties by increasing the concentration of the neurotransmitters serotonin and norepinephrine or indirectly acting against the overlapping tension-type headache to migraine [21].

Only a small percentage of group A participants was treated with BoNTA (19.4%) and even fewer patients with anti-CGRP MAbs (3%). The satisfaction with these treatments was rather modest, although tellingly, these findings were strongly biased to allow generalization. Specifically, for BoNTA, it was evident that about 40% of exposed patients were treated by other medical specialties, instead of neurologists/headache experts, with schemes not adhering to the approved fixed-site, fixed-dose (155–195 UI) PREEMPT protocol [22]. As such, it is strongly advised that experienced BoNTA injectors are required to guide and plan the therapeutic protocol for each patient with CM to optimize good and safe outcomes, based on widely acknowledged guidelines of BoNTA infusion for CM efficient prophylaxis [23]. Additionally, our findings on the efficacy and satisfaction of participants to anti-CGRP MAbs are not reliable and cannot be discussed because of strong bias imposed by the very limited use of this new category of prophylactic injectable medications in 2020, attributable to the un-approved use and lack of reimbursement for anti-CGRPs from the National Health System (NHS) and social services at that year. The formal market reimbursed release of anti-CGRPs after 2021 is expected to dramatically change the satisfaction of migraine patients to this modern prophylactic treatment. The exposure to external neurostimulation devices was also very limited (6.3%), in agreement with evidence supporting that generally Greek migraineurs seem to prefer oral medication to injection or stimulation devices for both prophylactic and symptomatic treatment of their migraine [24].

The findings on the exposure in CAMT are rather surprising to us, as it was evident that a rather high percentage (about 45%) of our participants received nutrients or was treated with homeopathy or acupuncture, although 80% of them discontinued these unapproved approaches due to ineffectiveness.

Regarding the use of acute treatments for the symptomatic relief of migraine attacks, it was evident that the majority of patients who were diagnosed and regularly followed by a neurologist/headache expert (group A) used triptans, as opposed to the much lower percentage of triptans use in group B patients (61.06% in group A vs. 18.08% in group Β). Moreover, group A patients seem to have comprehended the clinical benefits of early triptan use for the adequate symptomatic relief of migraine attack [25] as a significant percentage of them took triptans (62.5%) within 20 min after its onset.

As previously reported, although the data for this survey were collected during the COVID-19 pandemic, the majority of participants mentioned that they did not experience any effect on their migraine, including in treatment attitudes [16]. This is an intriguing finding given that an exacerbation in migraine severity was potentially anticipated as a result of SARS-CoV2 infection and vaccinations, which may cause a change in the nature or severity of headaches [26,27,28]. Indeed, there is evidence to support that anti-SARS-CoV2 vaccines are associated to a two-fold risk of developing headache with migraine-like features within 7 days from injection, likely due to systemic immunological reaction than to a vaccine-type specific reaction [29,30]. Post-vaccination headache incidence is considered to be higher in patients with a history of headache, including migraine, compared to naïve patients [31], while atypical phenomenology and course of migraine attacks may occasionally occur during COVID-19 infection [32]. Nonetheless, the intake of over-the-counter simple analgesics or NSAIDs, such as paracetamol, ibuprofen, and acetylsalicylic acid, might effectively alleviate the burden, which is associated with this temporary exacerbation of headache, particularly post-vaccination [33].

We should acknowledge as a limitation of our study the sampling process we applied to target advocacy group members with diagnosed migraine (group A) via a web-based survey and a questionnaire which is not validated or subjected to any form of psychometric evaluation, instead of a prospective follow up in our headache outpatients’ clinics. This approach restricted us from collecting more objective data. Moreover, the cross-sectional study design and the inclusion of participants with a tentative diagnosis of migraine at baseline (group B) may also further bias the interpretation of our findings.

## 5. Conclusions

Taken together, our results overall demonstrate that about 2 of 3 surveyed patients had received various prophylactic oral medications and the majority of them discontinued these prophylactic medications as a result of inefficacy/safety issues. BoNTA was reported to be effective only when administration was commenced by a trained neurologist/headache specialist, while our current findings are generally comparable to those obtained in our 2018 pre-COVID-19 survey [17] and the pandemic has not imposed any significant attitudes on migraine therapies and corresponding patients’ satisfaction. The pandemic and its restrictive measures imposed few barriers in health services access requiring a headache specialist or clinic, such as BoNTA injections for CM. Although a market change is anticipated with the evolving widespread use of anti-CGRPs MAbs or gepants in the symptomatic and prophylactic treatment of migraine, it is of great interest to review published results of larger longitudinal population-based studies to further ascertain the satisfaction of patients to migraine therapies both in our country and abroad.

## Data Availability

The corresponding author has full control of all primary data and agrees to allow the journal to review our data upon reasonable request.
